# Exposure to air pollution during preconceptional and prenatal periods and risk of hypertensive disorders of pregnancy: a retrospective cohort study in Seoul, Korea

**DOI:** 10.1186/s12884-018-1982-z

**Published:** 2018-08-22

**Authors:** Seung-Ah Choe, Yoon-Bae Jun, Sun-Young Kim

**Affiliations:** 1 Department of Obstetrics and Gynecology, School of Medicine, CHA University, Gyeonggi-do, Seongnam, 13488 South Korea; 2 Department of Epidemiology, Brown University School of Public Health, Providence, 02903 RI USA; 3 Department of Statistics, Seoul National University, Seoul, 08826 South Korea; 4 Department of Cancer Control and Population Health, Graduate School of Cancer Science and Policy, National Cancer Center, Gyeonggi-do, Goyang, 10408 South Korea

**Keywords:** Air pollution, Cohort, Gestational hypertension, Preconceptional exposure, Preeclampsia

## Abstract

**Background:**

Previous studies suggested associations between prenatal exposure to air pollution and hypertensive disorders of pregnancy. We explored the associations between ambient concentrations of five major air pollutants during preconceptional and prenatal periods and three hypertensive disorders of pregnancy in Seoul, Korea, using a population-representative cohort.

**Methods:**

We obtained heath and demographic data of pregnant women residing in Seoul for 2002–2013 from the Korean National Health Insurance Service–National Sample Cohort. For mother’s individual exposures to air pollution, we computed concentrations of particulate matter ≤10 μm in diameter (PM_10_), nitrogen dioxide (NO_2_), carbon monoxide (CO), sulfur dioxide (SO_2_), and ozone (O_3_) during 1, 3, 6, and 12 months to birth using regulatory monitoring data in Seoul. The associations between air pollution and hypertensive disorders were explored by using logistic regression models after adjusting for individual confounders.

**Results:**

Among 18,835 pregnant women in Seoul, 0.6, 0.5, and 0.4% of women developed gestational hypertension, preeclampsia, and preeclampsia requiring magnesium sulfate (Mg-preeclampsia), respectively. Although most odds ratios (ORs) were not statistically significant, we found increasing risk gradients with disease severity depending on the pollutant. There was the association between PM_10_ during 6 months to birth and gestational hypertension (OR for an interquartile range increase = 1.68 [95% confidence interval = 1.09–2.58]). NO_2_ and ozone during 12 and 1 month, respectively, before birth were associated with Mg-preeclampsia (1.43 [1.01–2.03], 1.53 [1.03–2.27]).

**Conclusions:**

We observed positive associations of exposure to some air pollutants before and during pregnancy with hypertensive disorders of pregnancy among the Korean general population. Future studies with refined exposure metrics should confirm our findings.

**Electronic supplementary material:**

The online version of this article (10.1186/s12884-018-1982-z) contains supplementary material, which is available to authorized users.

## Background

Hypertensive disorders of pregnancy are major direct causes of perinatal mortality and morbidity [1–3] leading to profound consequences on short-term and potentially long-term basis for maternal and child health [4, 5]. Hypertension developed during pregnancy in some women progress to preeclampsia and mothers with preeclampsia can experience fatal complications including eclampsia [6, 7]. The women with history of hypertensive disorders of pregnancy also have increased risk of cardiovascular disease, stroke, and type II diabetes later in life [8–11]. The babies of affected mothers are more likely to show intrauterine growth restriction, preterm birth, and low birth weight due to decreased placental blood supply and medically indicated delivery [12, 13]. Furthermore, the offspring shows higher rates of admission to neonatal intensive care units and increased risk of hospitalization for infectious, nervous, respiratory, endocrine, and metabolic complications [14]. In adulthood, they are more likely to have elevated blood pressure and increased risk of stroke [15, 16].

A growing body of evidence indicates that ambient air pollution is related to hypertensive disorders of pregnancy including preeclampsia [17–20]. Ambient concentrations of air pollutants such as particulate matter ≤10 and ≤ 2.5 μm in diameter (PM_10_ and PM_2.5_, respectively), nitrogen dioxide (NO_2_), carbon monoxide (CO), sulfur dioxide (SO_2_), and ozone (O_3_) were associated with hypertensive disorders of pregnancy [19, 21, 22] and with preterm delivery [23, 24]. Although the mechanism of this phenomenon remains unclear, it is hypothesized that air pollution could result in hypertensive disorders and placental hypoxia via vascular constriction, inflammation, and oxidative stress [20, 25]. However, the association between air pollution and hypertensive disorder in pregnancy has been inconsistent, depending on the susceptible period, severity of hypertensive disorder, and air pollutants of concern in previous studies [17, 21, 26]. This inconsistency may originate from generalizability of study population, unadjusted residual confounders, exposure assessment approach, and the different level of air pollution concentrations across study areas [20, 27]. In addition, most studies focused on air pollution exposure for a few months corresponding to each trimester, without consideration of possible pro-preeclamptic effect of preconceptional exposure.

The National Health Insurance Service–National Sample Cohort (NHIS-NSC) in Korea offers an opportunity for exploring the relationship between air pollution and hypertensive disorders of pregnancy among the general population. This cohort provides clinical risk factors and disease diagnoses in addition to general demographics. We restricted our analysis to Seoul, the capital of South Korea, which is densely populated (~ 10 million people) and highly polluted (annual average concentration of PM_10_ = 51.3 μg/m^3^ in 2010) compared to the World Health Organization guideline (PM_10_ = 20 μg/m^3^) [28]. Previous epidemiological studies in Seoul reported associations between air pollution and adverse birth outcomes [29–31]. Herein, we aimed to explore the association between three types of hypertensive disorders of pregnancy and exposure to five primary pollutants (PM_10,_ NO_2_, CO, SO_2_, and O_3_) before and during pregnancy in Seoul using the NHIS-NSC data.

## Methods

### Study population

This study was based on the NHIS-NSC data which can be accessed through the National Health Insurance Data Sharing Service website (http://nhiss.nhis.or.kr). Applicants can gain access to NHIS-NSC data after securing institutional review board (IRB) approvals and making payments [32]. The Korean National Health Insurance (NHI) is a single-insurer system established in 1989 that covers all nationals and eligible immigrants who reside in South Korea [33]. Its database contains general demographics, including household income in percentiles and residential address, and clinical information on diagnoses, prescribed medications, procedures, and treatments covered by NHI for every visit to a health institution. From this database, the NHIS created the NHIS-NSC of approximately 1 million Korean participants who were sampled in 2002 based on a population-representative sampling design and followed for 11 years unless participants were disqualified due to death or emigration. Individuals and health institutions in the cohort were anonymized. The detailed cohort profile is previously published [32]. Over 100 studies of medical conditions such as cardiovascular diseases and diabetes have been published using the NHIS-NSC data, since it became publicly available in 2014 [34–38].

We identified women in the NHIS-NSC who gave birth using the following stepwise inclusion criteria based on the hierarchical method suggested by Kuklina et al. and applied to previous Korean studies [34, 35, 39]: 1) those with an obstetric treatment history in an obstetrics and gynecology department and with a diagnostic code for pregnancy-related conditions (starting with ‘O8’ in the International Statistical Classification of Disease and Related Health Problems, 10th Revision [ICD-10]); 2) those with hospital admission made under a treatment code for child birth; and 3) those aged between 15 and 44 years. For women with multiple births, we included only the earliest births in the cohort. Finally, women who lived in Seoul were selected for the analysis.

### Maternal exposure assessment

Hourly measurements of PM_10_, NO_2_, CO, SO_2_, and O_3_ at about 40 air quality monitoring sites in Seoul from 2001 through 2013 were obtained from the National Institute of Environmental Research (NIER). We used urban background sites, after excluding urban roadside sites, to represent residential exposures. Each of the 25 districts (the area of 10–47 km^2^) in Seoul has had one urban background site over the entire study period. For the districts with more than one site run for a limited period, we used the site operated for the entire period. Using hourly measurements of these air pollutants, we calculated daily representative concentrations of five pollutants: 24-h averages for PM_10_, NO_2_, and SO_2_, and maximum of 17 8-h moving averages for CO and O_3_. These maximum concentrations of CO and O_3_ reflected the high concentrations during commuting hours and high correlation with sunlight [40].

We computed individual exposures of each mother by four exposure periods. Since date of birth was available without gestational age at birth in the NHIS-NSC data, we defined the four exposure periods as 12, 6, 3, and 1 month to birth, instead of each trimester. Whereas 6- to 1-month exposure represents trimester-based exposure during pregnancy, 12-month exposure additionally incorporates preconceptional period [41, 42]. We averaged daily concentrations for each of the four periods to derive representative exposure estimates for the corresponding period. Since the residential addresses were available at the district level, we assigned district-averages to mothers living in the same district as individual exposures. We assumed that mothers’ addresses at the time of childbirth were consistent to those during 12, 6, 3, and 1 month to birth, given low moving rate within a year in the cohort [43].

### Confounding variables

We included established risk factors in the model for exploring the relationship between air pollution and hypertensive disorders of pregnancy. The risk factors were age, parity, plurality, paid employment, presence of diabetes (gestational or non-gestational) and relative level of household income [1, 3, 44]. The household income was classified into three groups (low, 0–40%; middle, 40–80%; and high, 80–100%). Diagnosis of diabetes were determined by any type of diabetes based on ICD-10 codes (E10, type 1 diabetes mellitus; E11, type 2 diabetes mellitus; E12, malnutrition-related diabetes mellitus; E13, other specified diabetes mellitus; E14, unspecified diabetes mellitus; and O24, diabetes mellitus in pregnancy). When these codes coexisted with obstetric delivery in the same year, the case was determined to have the corresponding condition.

Hypertensive disorders of pregnancy were grouped into three categories representing mild to severe disease: gestational hypertension, preeclampsia, and preeclampsia requiring magnesium sulfate (Mg-preeclampsia). To reflect severity of preeclampsia, the three hypertensive conditions were determined with corresponding ICD-10 and procedure codes. Gestational hypertension was defined as the presence of ICD-10 codes for hypertension in pregnancy (O13, gestational hypertension without significant proteinuria) excluding preeclampsia cases. Presence of ICD-10 codes for preeclampsia (O14.0, mild-to-moderate preeclampsia; O14.1, severe preeclampsia; O14.2, HELLP (hemolysis, elevated liver enzymes, and lower platelets) syndrome; and O14.9, preeclampsia, unspecified) without the procedure code for intravenous magnesium sulfate (Magnesium sulfate) infusion was determined to be preeclampsia to represent cases with mild to moderate severity. Magnesium sulfate is used as the first-line treatment for severe preeclampsia presented with any symptom or sign of impending eclampsia such as blurred vision, upper abdominal pain and hyperreflexia [45]. Since magnesium sulfate can be used for other indication than preeclampsia, Mg-preeclampsia was defined as a condition with coexistence of the ICD-10 code for preeclampsia and procedure code for magnesium sulfate infusion.

### Statistical analysis

The individual characteristics and concentrations of the five air pollutants during the four periods were compared between any pair of the normotensive (no hypertensive disorders of pregnancy) and the three hypertensive disorder groups (gestational hypertension, preeclampsia, and Mg-preeclampsia). Pearson’s Chi-squared test with Yates’ continuity correction was used for paired-comparison of socio-demographic and clinical characteristics. Student t test was used for comparison of averaged concentrations of the five air pollutants. To examine the association between air pollution and gestational hypertensive disorder, we conducted logistic regression analysis for each pair of the five pollutants and three hypertensive disorder outcomes by the four periods. The reference group for analyses of all three hypertensive disorder outcomes was the normotensive. In the model, we adjusted for maternal age, household income, paid employment, nulliparity, plurality, diabetes, and a long-term temporal trend. The temporal trend representing seasonal and annual changes of hypertensive disorders of pregnancy was characterized by using natural cubic spline with 12 degrees of freedom (df) (1 df per year). Effect estimates were presented with odds ratios (ORs) and 95% confidence intervals (CIs) for interquartile range (IQR) increases in each of the five pollutant concentrations. We conducted several sensitivity analyses including temporal adjustment with different degrees of freedom and adding interaction of the four seasons on hypertensive disorders. The four seasons of spring, summer, autumn, and winter were defined as March–May, June–August, September–November, and December–February, respectively. The analyses were performed using R (ver. 3.0.3; R Development Core Team, Vienna, Austria).

This study was reviewed and approved by the Institutional Review Board of Gangnam CHA hospital (IRB No. GCI-16-22).

## Results

In NHIS-NSC, 18,835 women who lived in Seoul at the time of delivery were identified from 2002 through 2013. The prevalences of hypertensive disorders of pregnancy were 5.7, 4.7, and 4.1 per 1000 pregnant women for gestational hypertension, preeclampsia, and Mg-preeclampsia, respectively. Table 1 summarizes the basal characteristics of the normotensive and three hypertensive disorder groups. Majority of women were aged 25–34 years (78%), housewives (71%) and nulliparous (60%). There was no Mg-preeclampsia case in women aged 15–24 years. The gestational hypertension group had higher proportion of women with advanced age than the normotensive group. Proportions of nulliparity, multiple gestation, and diabetes were higher in women with preeclampsia and Mg-preeclampsia than in the normotensive. The normotensive group showed higher level of household income than the gestational hypertension group; however, the composition in the household income tertiles was not different among the normotensive and each of the preeclampsia and Mg-preeclampsia groups. No significant seasonality was observed in the birth date of the normotensive and three hypertensive groups. Average PM_10_ and NO_2_ concentrations during 12 months to birth were lower in the gestational hypertension group than those in the normotensive. Concentrations of all air pollutants except SO_2_ during 12 months to birth were higher in the Mg-preeclampsia group compared with the gestational hypertension group. In general, correlation coefficients for average concentrations of five air pollutants during the four exposure periods were moderate to high (*r* = 0.43–0.85) between 6 and 3 months and between 3 and 1 month to birth (Table 2).

**Table 1:** Characteristics and five air pollutant concentrations of normotensive, gestational hypertension, preeclampsia and Mg-preeclampsia groups in 18,835 pregnant women residing in Seoul for 2002–2013 from the Korean National Health Insurance Service–National Sample Cohort

Characteristics	Normotensive group *N* = 18,565	Gestational hypertension *N* = 105	Preeclampsia *N* = 88	Mg-preeclampsia *N* = 77
*Maternal age (years)*				
15–24	542 (2.9)	3 (2.8)	4 (4.5)	0 (0)
25–34	14,564 (78.4)	70 (66.7)^a^	65 (73.9)	57 (74.0)
35–44	3459 (18.7)	32 (30.5)^a^	19 (21.6)	20 (26.0)
Paid employment	5309 (28.6)	32 (30.5)	23 (26.1)	22 (28.6)
Nulliparity	10,551 (56.8)	69 (65.7)	67 (76.1)^a^	53 (68.8)^a^
Multiple gestation	268 (1.4)	2 (1.9)	7 (8.0)^a^	7 (9.1)^a, b^
Diagnosis of diabetes	297 (1.6)	8 (7.6)^a^	7 (8.0)^a^	7 (9.1)^a^
*Household income (%)*				
0–40	3509 (18.9)	27 (25.7)^a^	14 (15.9)	16 (20.8)
40–70	7607 (41.0)	51 (48.6)^a^	42 (47.7)	38 (49.3)
70–100	7449 (40.1)	27 (25.7)^a^	32 (36.4)	23 (29.9)
*Birth season*				
Spring	4689 (25.3)	34 (32.4)	22 (25.0)	22 (28.6)
Summer	4288 (23.1)	22 (21.0)	17 (19.3)	23 (29.9)
Fall	4916 (26.5)	27 (25.7)	30 (34.1)	18 (23.3)
Winter	4672 (25.1)	22 (21.0)	19 (21.6)	14 (18.2)
PM_10_ (μg/m^3^) for four exposure periods				
12 months to birth	57.78 ± 10.31	54.72 ± 10.40^a^	58.08 ± 11.57	58.15 ± 9.81^b^
6 months	56.23 ± 13.56	54.11 ± 13.75	54.64 ± 14.11	55.84 ± 13.22
3 months	56.34 ± 16.68	54.15 ± 15.58	52.72 ± 14.80^a^	56.56 ± 16.99
1 month	56.68 ± 20.40	53.12 ± 16.58	52.95 ± 17.79 ^a^	53.40 ± 17.64
NO_2_ (ppb)				
12 months	35.33 ± 4.42	34.10 ± 4.51^a^	35.07 ± 4.97	36.31 ± 4.38^b^
6 months	35.12 ± 5.85	33.99 ± 5.38^a^	34.51 ± 6.27	36.02 ± 5.84^b^
3 months	35.19 ± 7.22	34.30 ± 6.50	34.41 ± 7.09	35.43 ± 7.82
1 month	35.33 ± 8.47	33.70 ± 7.07^a^	35.13 ± 7.54	34.62 ± 9.52
CO (100 ppb)				
12 months	76.16 ± 14.45	73.13 ± 13.80^a^	77.34 ± 16.43	77.65 ± 12.13^b^
6 months	75.20 ± 18.93	72.23 ± 17.41	75.42 ± 19.83	77.27 ± 19.58
3 months	75.28 ± 23.28	72.72 ± 19.94	74.41 ± 22.76	76.03 ± 23.92
1 month	75.24 ± 25.85	70.69 ± 21.54	73.12 ± 23.17	74.24 ± 29.24
SO_2_ (ppb)				
12 months	5.25 ± 1.06	5.12 ± 0.90	5.35 ± 1.10	5.18 ± 1.09
6 months	5.24 ± 1.43	5.11 ± 1.24	5.20 ± 1.47	5.31 ± 1.57
3 months	5.27 ± 1.74	5.26 ± 1.53	5.21 ± 1.81	5.17 ± 1.77
1 month	5.29 ± 1.99	5.17 ± 1.56	5.35 ± 1.96	4.97 ± 2.15
O_3_ (ppm)				
12 months	29.74 ± 4.93	31.33 ± 4.28	29.51 ± 5.11^b^	29.49 ± 5.21^b^
6 months	29.54 ± 8.90	29.87 ± 8.39	29.56 ± 9.67	28.55 ± 9.12
3 months	29.36 ± 11.40	29.62 ± 10.46	28.20 ± 10.76	30.67 ± 11.98
1 month	29.54 ± 12.86	30.37 ± 11.92	27.71 ± 11.04	31.70 ± 13.94

*PM*_*10*_ particulate matter, *NO*_*2*_ nitrogen dioxide, *CO* carbon monoxide, *SO*_*2*_ sulfur dioxide, *O*_*3*_ ozone

Numbers presented in parentheses are percentages. Concentrations of air pollutants are shown as mean ± standard deviation. Pearson’s Chi-squared test with Yates’ continuity correction was used for comparison between pairs of normotensive and three hypertensive disorder groups. Student *t* test was used for comparison of period-average concentrations of the five air pollutants between pairs of normotensive and three hypertensive disorder groups

^a^Significant difference (*p* < .05) compared with the normotensive group. ^b^Significant difference (*p* < .05) compared with the gestational hypertension group. No significant differences between preeclampsia and Mg-preeclampsia groups

**Table 2:** Pearson correlation coefficients between pairs of period-average concentrations of five air pollutants in 18,835 pregnant women residing in Seoul for 2002–2013 from the Korean National Health Insurance Service–National Sample Cohort

Exposure period	12 months	6 months	3 months
PM_10_			
6 months	0.74	–	–
3 months	0.61	0.8	–
1 month	0.5	0.53	0.8
NO_2_			
6 months	0.77	–	–
3 months	0.62	0.82	–
1 month	0.51	0.56	0.82
CO			
6 months	0.69	–	–
3 months	0.53	0.81	–
1 month	0.43	0.51	0.85
SO_2_			
6 months	0.74	–	–
3 months	0.58	0.83	–
1 month	0.49	0.56	0.85
O_3_			
6 months	0.57	–	–
3 months	0.43	0.77	–
1 month	0.35	0.43	0.84

*PM*_*10*_ particulate matter, *NO*_*2*_ nitrogen dioxide, *CO* carbon monoxide, *SO*_*2*_ sulfur dioxide, *O*_*3*_ ozone

**Fig. 1:**
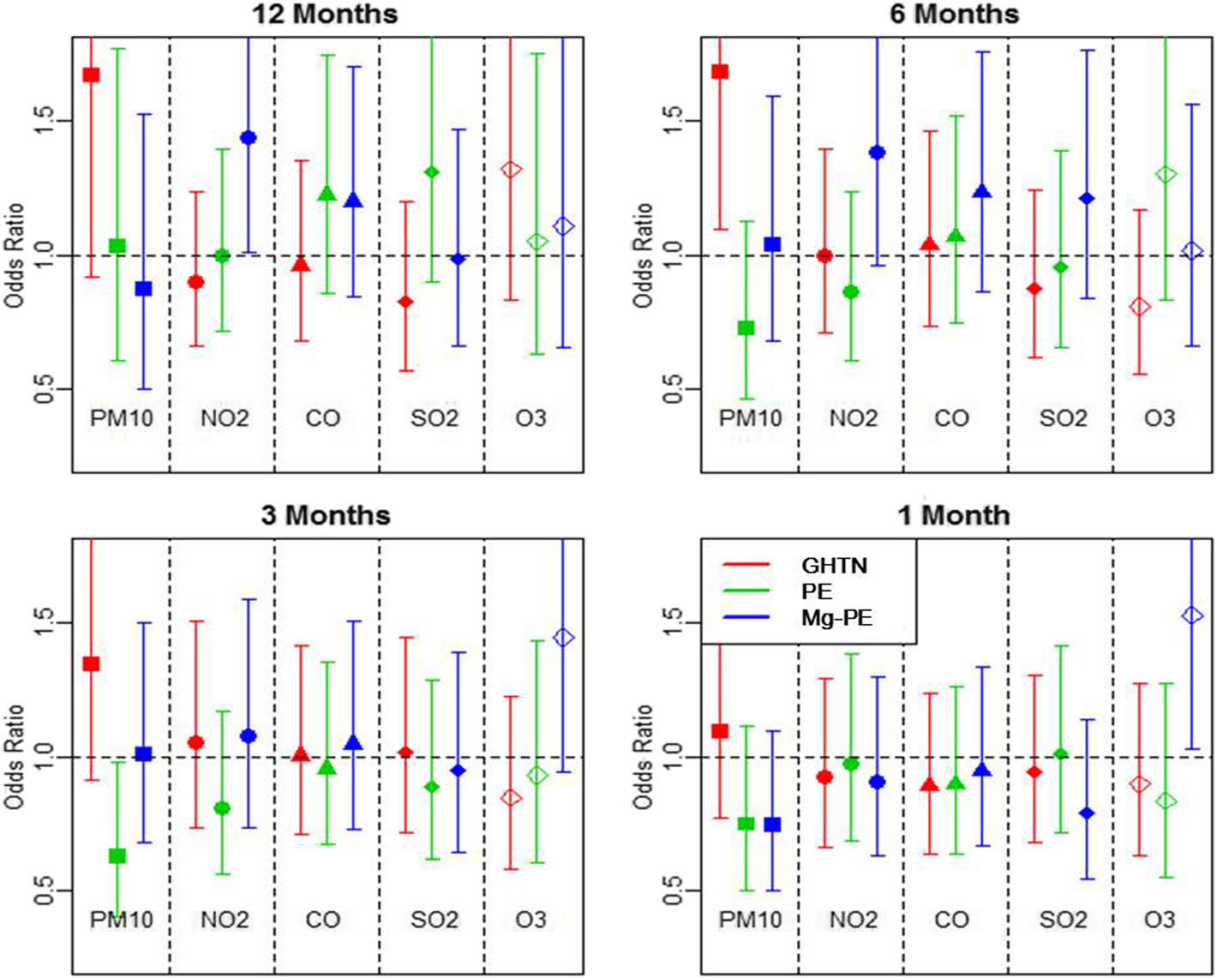
Odds ratios and 95% confidence intervals of three types of hypertensive disorders of pregnancy for interquartile range increases in five air pollutant concentrations by four exposure periods in 18,835 pregnant women residing in Seoul from the Korean National Health Insurance Service–National Sample Cohort for 2002–2013. PM_10,_ particulate matter; NO_2,_ nitrogen dioxide; CO, carbon monoxide, SO_2,_ sulfur dioxide; O_3,_ ozone; GHTN, Gestational hypertension; PE, preeclampsia; Mg-PE, preeclampsia requiring magnesium sulfate. Date of birth was adjusted using non-parametric cubic spline smoothing (degree of freedom = 12)

Adjusted risk estimates of gestational hypertension, preeclampsia, and Mg-preeclampsia for IQR increases of the five air pollutants are summarized in Fig. 1. Patterns of the associations varied by pollutants, hypertensive disorders, and exposure periods; however, most risk estimates were not statistically significant. Average concentrations of PM_10_ during 12, 6, and 3 months before birth were significantly or marginally associated with gestational hypertension (OR = 1.67 [95% CI, 0.91–3.04], 1.68 [1.09–2.58], and 1.35 [0.91–1.99], respectively). There was no association with preeclampsia or Mg-preeclampsia. For NO_2,_ CO, and SO_2_ during 12 and 6 months before birth, the risk estimate for each hypertensive disorder generally increased with disease severity though the findings were close to null. Average concentration of NO_2_ during 12 months to birth was associated with Mg-preeclampsia (OR = 1.44 [95% CI, 1.01, 2.05]). Compared to the other four pollutants, O_3_ showed increasing risk for a shorter exposure period. Increasing average concentration of O_3_ for 1 month before birth was associated with Mg-preeclampsia (OR = 1.53 [95% CI, 1.03–2.27]).

In our sensitivity analysis, no particular season showed consistently large risk estimates compared to the other seasons (Additional file 1: Figure S1). Patterns of risk estimates using different degrees of freedom, which reflects various temporal adjustment, were generally consistent with those of primary analyses (see Additional file 2: Figure S2).

## Discussion

Using a population-representative cohort, this study investigated patterns of associations between concentrations of five major air pollutants and three hypertensive disorders of pregnancy for four exposure periods. Although mostly null risk estimates were observed, there were generally increasing risk gradients for all pollutants, except for PM_10_, to more severe forms of hypertensive disorders of pregnancy. Risk estimates were higher with longer exposure periods than shorter periods, except for O_3_. In particular, this study suggested an association between air pollution during preconceptional period and preeclampsia which has not been explored previously.

In this study, we observed patterns of higher risk estimates for hypertensive disorders of pregnancy with longer exposure periods such as 12 or 6 months than with shorter exposure periods, except for O_3_. Most studies suggested that the pro-hypertensive effect of air pollution would be short-term (i.e., 2nd or 3rd trimester) rather than long-term (12 months before birth including the preconceptional period). Thus, this finding may need another explanation. During the preconceptional period, exposure to high concentrations of air pollutants would induce systemic inflammatory reactions, which would have mediated changes in the vascular bed and blood pressure in normal healthy individuals [46, 47]. However, an adaptive process to increased inflammatory cytokines and vascular resistance in pregnant women may suppress immediate changes in blood pressure [48]. As blood pressure in pregnancy gradually increases until child birth [22], exposure to increased air pollutants long before birth would make women more vulnerable to preeclampsia. Especially for NO_2_ exposure during 12 months before birth, this change might have occurred in a cumulative manner, showing generally greater risks with disease severity as shown in this study. The cumulative adverse effect of NO_2_ was consistent with previous findings in normal non-pregnant individuals [18, 49]. Thus, the reason for the null findings for most air pollutants during pregnancy for short terms may be due to the relatively short period to provoke hypertensive or preeclamptic change in this study population. The differential impact of these air pollutants on hypertensive disorders should be further clarified in future studies.

We found positive associations of PM_10_ with gestational hypertension, but not with preeclampsia or Mg-preeclampsia, which is consistent with a previous study [17]. Some previous studies of relationships between PM_10_ exposure during pregnancy and gestational hypertension have shown notable heterogeneity [17, 22, 50]. Our study also showed a negative association between PM_10_ and preeclampsia that we did not anticipate. Based on the general pattern of no associations for PM_10_ and preeclampsia, it might be one of the random findings which can be occurred in multiple simultaneous testing [51]. Also different levels of PM_10_ concentrations or characteristics of study population would have contributed to the inconsistency. The different associations between three pregnancy-related hypertensive disorders and air pollution may reflect their different disease entities with distinct etiology and mechanism [52]. Potential factors underlying the different associations between hypertensive conditions and settings should be confirmed with well-designed studies in the future.

This study showed non-significant risk estimates for most pairs of air pollutants, hypertensive disorders, and exposure periods. Relatively crude estimates for individual exposures could have resulted in exposure measurement error and subsequent null associations. We used the district-average concentrations as individual exposures, which do not represent the spatial variability of air pollution among individuals within the same district. Recent epidemiological studies of air pollution assessed individual-level exposure to air pollution by using fine-scale address data and exposure prediction approaches such as land use regression, air quality models, and geostatistical methods [53–55]. These models included a large suite of geographic variables, satellite image data, or spatial correlation structure to better represent spatial variability of air pollution across people’s homes. However, because residential addresses in NHIS-NSC are available at the district level without fine spatial-scale information such as towns and streets, we were not able to apply these modeling approaches. The refined exposure assessment using fine-scale address data may yield more precise and/or accurate risk estimates. However, this exposure misclassification would not be correlated with outcome and could have led to risk estimates toward the null [56]. Thus, true effects may be higher than those observed in this study. In addition, our use of consistent exposure estimates during pregnancy based on residential addresses at the time of birth without incorporating moving would have resulted in the null findings [20]. However, this impact could be minimal given low moving rate within a year in the same cohort [43].

Prevalence of preeclampsia and Mg-preeclampsia (0.9%) in our study population is much lower than those in other countries. Prevalence of preeclampsia was 2.6–3.4% in US women [20, 57] and 1.2–6% in European population [17, 26, 58]. If patients of preeclampsia or Mg-preeclampsia were not coded in the NHIS database and treated as normal cases in our analysis, this misclassification would have resulted in underestimation of the prevalence of preeclampsia. However, under-reporting is less likely in health insurance claim data created based on the national health insurance system that requires diagnosis codes for reimbursement in South Korea [59]. There are at least three possible explanations for the low prevalence in this study: Asian ethnicity, metropolitan residential environments, and early live birth status. First, some studies reported low rates of preeclampsia in Asian population. A study that compared preeclampsia across different race/ethnicity populations showed the lowest rate of mild and severe preeclampsia in Asian/Pacific Islanders (2.0% and 1.6%) and the highest in non-Hispanic black (4.8% and 3.5%) [60]. Other studies reported that severe preeclampsia was developed in 0.49% of Chinese women [61], 0.5% in Danish [62], and 0.4% in British study [63]. Second, because women living in Seoul, the capital of South Korea, showed generally better health conditions than others in small cities or rural areas [64, 65], our restriction of the study population to those living in Seoul possibly resulted in low hypertensive disorders of pregnancy compared to other reports. In our previous studies for South Korea, prevalence of preeclampsia in total and severe preeclampsia (requiring MgSO_4_) was higher (1.2 and 0.9%) than ours [34, 35]. A Chinese study also showed significant geographical differences in the prevalence of hypertensive disorders, showing the highest (7.44%) in Northern China and the lowest (1.23%) in Central China [66]. Lastly, inclusion of only one live birth case for each mother might have further lowered the prevalence by excluding recurrent preeclampsia and deliveries ended with stillbirth. These three explanations could also be applied to low prevalence of gestational hypertension found in our study. In addition, previous studies of gestational hypertension prevalence showed large variation across population and study areas: low rates of 1.2% in Central China [66] and 1.4% in New York City [67], and high rates of 19.4% in Zimbabwe [68] and 11.6% in Nigeria [69].

This study has several additional limitations. The risk estimates for 6, 3, and 1 month to birth in this study cannot be directly compared with those in previous studies which were calculated for the first or second trimester of pregnancy. However, as the sign of preeclampsia or severe preeclampsia became evident beyond the second trimester in most cases, the gestational age corresponding to 3 or 1 month to birth may not be critically different from that of the second trimester. The null result for the relationship between air pollution during 1 or 3 months to birth and hypertensive disorders of pregnancy except O_3_ and Mg-preeclampsia is consistent with the results of several studies that reported same null findings in the second trimester [19, 20]. Second, we were not able to assess and control for the influence of important confounders such as smoking or obesity before and during pregnancy. Because the prevalence of smokers would be extremely low in pregnant Korean women [70] and presence of diabetes (which is closely related with obesity) was included in our model, results with these confounders would not be significantly changed. Lastly, this study did not include PM_2.5_ because there were limited available data for PM_2.5_ before 2008 in Seoul. PM_2.5_ has been suggested to have a greater health impact than PM_10_ [71]. With longer temporal data, the associations between PM_2.5_ and hypertensive disorders of pregnancy should be explored in future studies_._

## Conclusions

This study suggests positive associations between exposure to some air pollutants during preconceptional and prenatal periods and hypertensive disorders of pregnancy among the general population in Seoul. Future studies with refined exposure metrics are needed to confirm our findings.

## Additional files

Additional file 1: **Figure S1.** Odds ratios and 95% confidence intervals of three types of hypertensive disorders of pregnancy for interquartile range increases in five air pollutant concentrations for 12 months and 1 month before birth by four birth seasons in 18,835 pregnant women residing in Seoul from the Korean National Health Insurance Service–National Sample Cohort for 2002–2013. No particular season showed consistently large risk estimates compared to the other seasons. PM_10,_ particulate matter; NO_2,_ nitrogen dioxide; CO, carbon monoxide, SO_2,_ sulfur dioxide; O_3,_ ozone; GHTN, Gestational hypertension; PE, preeclampsia; Mg-PE, preeclampsia requiring magnesium sulfate. First hollow squares indicate risk estimates not adjusted for birth season. From the first to fourth solid squares in the same color represent spring, summer, fall and winter in order. (DOCX 142 kb)

Additional file 2: **Figure S2.** Odds ratios and 95% confidence intervals of three types of hypertensive disorders of pregnancy for interquartile range increases in five air pollutant concentrations for 12 months and 1 month before birth using different degrees of freedom in adjustment for a temporal trend. Patterns of risk estimates using different degrees of freedom were generally consistent with those of primary analyses. (DOCX 127 kb)

## Abbreviations

CO: Carbon monoxide
df: Degrees of freedom
HELLP: Hemolysis, elevated liver enzymes, and lower platelets
ICD: International classification of disease
NO_2_: Nitrogen dioxide
O_3_: Ozone
OR: Odds ratios
PM_10_: Particulate matter ≤10 μm in diameter
PM_2.5_: Particulate matter ≤2.5 μm in diameter
SO_2_: Sulfur dioxide

## References

[1] Roberts JM, Redman CW (1993). Pre-eclampsia: more than pregnancy-induced hypertension. Lancet.

[2] Roberts JM, Cooper DW (2001). Pathogenesis and genetics of pre-eclampsia. Lancet.

[3] Roberts JM (1996). Preventing pre-eclampsia. Lancet.

[4] Ananth CV, Basso O (2010). Impact of pregnancy-induced hypertension on stillbirth and neonatal mortality in first and higher order births: a population-based study. Epidemiology.

[5] Endeshaw G, Berhan Y (2015). Perinatal outcome in women with hypertensive disorders of pregnancy: a retrospective cohort study. Int Sch Res Notices.

[6] Mattar F, Sibai BM (2000). Eclampsia. VIII. Risk factors for maternal morbidity. Am J Obstet Gynecol.

[7] Mammaro A, Carrara S, Cavaliere A, Ermito S, Dinatale A, Pappalardo EM (2009). Hypertensive disorders of pregnancy. J Prenat Med.

[8] Umesawa M, Kobashi G (2017). Epidemiology of hypertensive disorders in pregnancy: prevalence, risk factors, predictors and prognosis. Hypertens Res.

[9] Heida KY, Franx A, van Rijn BB, Eijkemans MJ, Boer JM, Verschuren MW (2015). Earlier age of onset of chronic hypertension and type 2 diabetes mellitus after a hypertensive disorder of pregnancy or gestational diabetes mellitus. Hypertension.

[10] Feig DS, Shah BR, Lipscombe LL, Wu CF, Ray JG, Lowe J (2013). Preeclampsia as a risk factor for diabetes: a population-based cohort study. PLoS Med.

[11] Wilson BJ, Watson MS, Prescott GJ, Sunderland S, Campbell DM, Hannaford P (2003). Hypertensive diseases of pregnancy and risk of hypertension and stroke in later life: results from cohort study. BMJ.

[12] Barton JR, O'Brien JM, Bergauer NK, Jacques DL, Sibai BM (2001). Mild gestational hypertension remote from term: progression and outcome. Am J Obstet Gynecol.

[13] Buchbinder A, Sibai BM, Caritis S, Macpherson C, Hauth J, Lindheimer MD (2002). Adverse perinatal outcomes are significantly higher in severe gestational hypertension than in mild preeclampsia. Am J Obstet Gynecol.

[14] Backes CH, Markham K, Moorehead P, Cordero L, Nankervis CA, Giannone PJ: Maternal preeclampsia and neonatal outcomes. J Pregnancy 2011, 2011:214365. 10.1155/2011/214365 PMC3087144 21547086

[15] Yesil GD, Gishti O, Felix JF, Reiss I, Ikram MK, Steegers EA (2016). Influence of maternal gestational hypertensive disorders on microvasculature in school-age children: the generation R study. Am J Epidemiol.

[16] Huckstep O, Lewandowski AJ, Leeson P (2016). Invited commentary: hypertension during pregnancy and offspring microvascular structure-insights from the retinal microcirculation. Am J Epidemiol.

[17] van den Hooven EH, de Kluizenaar Y, Pierik FH, Hofman A, van Ratingen SW, Zandveld PY (2011). Air pollution, blood pressure, and the risk of hypertensive complications during pregnancy: the generation R study. Hypertension.

[18] Coogan PF, White LF, Jerrett M, Brook RD, Su JG, Seto E (2012). Air pollution and incidence of hypertension and diabetes in African American women living in Los Angeles. Circulation.

[19] Xu X, Hu H, Ha S, Roth J (2014). Ambient air pollution and hypertensive disorder of pregnancy. J Epidemiol Community Health.

[20] Savitz DA, Elston B, Bobb JF, Clougherty JE, Dominici F, Ito K (2015). Ambient fine particulate matter, nitrogen dioxide, and hypertensive disorders of pregnancy in new York City. Epidemiology.

[21] Hu H, Ha S, Roth J, Kearney G, Talbott EO, Xu X (2014). Ambient air pollution and hypertensive disorders of pregnancy: a systematic review and meta-analysis. Atmos Environ (1994).

[22] Pedersen M, Stayner L, Slama R, Sorensen M, Figueras F, Nieuwenhuijsen MJ (2014). Ambient air pollution and pregnancy-induced hypertensive disorders: a systematic review and meta-analysis. Hypertension.

[23] Wu J, Ren C, Delfino RJ, Chung J, Wilhelm M, Ritz B (2009). Association between local traffic-generated air pollution and preeclampsia and preterm delivery in the south coast air basin of California. Environ Health Perspect.

[24] Li S, Guo Y, Williams G. Acute impact of hourly ambient air pollution on preterm birth. Environ Health Perspect. 2016; 10.1289/EHP200 PMC5047774 27128028

[25] Veras MM, Damaceno-Rodrigues NR, Caldini EG, Maciel Ribeiro AA, Mayhew TM, Saldiva PH (2008). Particulate urban air pollution affects the functional morphology of mouse placenta. Biol Reprod.

[26] Dadvand P, Figueras F, Basagana X, Beelen R, Martinez D, Cirach M (2013). Ambient air pollution and preeclampsia: a spatiotemporal analysis. Environ Health Perspect.

[27] Ritz B, Wilhelm M (2008). Ambient air pollution and adverse birth outcomes: methodologic issues in an emerging field. Basic Clin Pharmacol Toxicol.

[28] WHO: WHO Air quality guidelines for particulate matter, ozone, nitrogen dioxide and sulfur dioxide - Global update 2005. In: WHO Air quality guidelines. WHO Press, World Health Organization; 2006. 34662007

[29] Seo JH, Leem JH, Ha EH, Kim OJ, Kim BM, Lee JY (2010). Population-attributable risk of low birthweight related to PM10 pollution in seven Korean cities. Paediatr Perinat Epidemiol.

[30] Yi S-J, Kim H, Kim S-Y (2016). Exploration and application of regulatory PM_10_ measurement data for developing long-term prediction models in South Korea. J Korean Soc Atmos Environ.

[31] Ha EH, Hong YC, Lee BE, Woo BH, Schwartz J, Christiani DC (2001). Is air pollution a risk factor for low birth weight in Seoul?. Epidemiology.

[32] Lee J, Lee JS, Park SH, Shin SA, Kim K (2017). Cohort profile: the National Health Insurance Service-National Sample Cohort (NHIS-NSC)**,** South Korea. Int J Epidemiol.

[33] Kwon S (2003). Payment system reform for health care providers in Korea. Health Policy Plan.

[34] Choe SA, Min HS, Cho SI (2017). Decreased risk of preeclampsia after the introduction of universal voucher scheme for antenatal care and birth Services in the Republic of Korea. Matern Child Health J.

[35] Choe SA, Min HS, Cho SI (2016). The income-based disparities in preeclampsia and postpartum hemorrhage: a study of the Korean National Health Insurance cohort data from 2002 to 2013. Springerplus.

[36] Kim NH, Kwon TY, Yu S, Kim NH, Choi KM, Baik SH (2017). Increased vascular disease mortality risk in Prediabetic Korean adults is mainly attributable to ischemic stroke. Stroke.

[37] Choi JB, Moon HW, Park YH, Bae WJ, Cho HJ, Hong SH (2016). The impact of diabetes on the risk of prostate Cancer development according to body mass index: a 10-year Nationwide cohort study. J Cancer.

[38] Noh J, Han KD, Ko SH, Ko KS, Park CY (2017). Trends in the pervasiveness of type 2 diabetes, impaired fasting glucose and co-morbidities during an 8-year-follow-up of nationwide Korean population. Sci Rep.

[39] Kuklina EV, Whiteman MK, Hillis SD, Jamieson DJ, Meikle SF, Posner SF (2008). An enhanced method for identifying obstetric deliveries: implications for estimating maternal morbidity. Matern Child Health J.

[40] Kim SY, O'Neill MS, Lee JT, Cho Y, Kim J, Kim H (2007). Air pollution, socioeconomic position, and emergency hospital visits for asthma in Seoul, Korea. Int Arch Occup Environ Health.

[41] Baiz N, Slama R, Bene MC, Charles MA, Kolopp-Sarda MN, Magnan A (2011). Maternal exposure to air pollution before and during pregnancy related to changes in newborn's cord blood lymphocyte subpopulations. The EDEN study cohort. BMC Pregnancy Childbirth.

[42] Rappazzo KM, Daniels JL, Messer LC, Poole C, Lobdell DT (2014). Exposure to fine particulate matter during pregnancy and risk of preterm birth among women in New Jersey, Ohio, and Pennsylvania, 2000-2005. Environ Health Perspect.

[43] Kim O, Kim S-Y, Kwon H-Y, Kim H (2017). Data issues and suggestions in the National Health Insurance Service-National Sample Cohort for assessing the association between long-term exposure to air pollution and mortality. J Health Info Stat.

[44] Subramaniam V (2007). Seasonal variation in the incidence of preeclampsia and eclampsia in tropical climatic conditions. BMC Womens Health.

[45] WHO, UNICEF, UNPF (2017). Managing complications in pregnancy and childbirth: a guide for midwives and doctors – 2nd ed.

[46] Robledo CA, Mendola P, Yeung E, Mannisto T, Sundaram R, Liu D (2015). Preconception and early pregnancy air pollution exposures and risk of gestational diabetes mellitus. Environ Res.

[47] Zhang J, Zhu T, Kipen H, Wang G, Huang W, Rich D, et al. Cardiorespiratory biomarker responses in healthy young adults to drastic air quality changes surrounding the 2008 Beijing Olympics. Res Rep Health Eff Inst. 2013(174):5–174. PMC4086245 23646463

[48] Lee PC, Roberts JM, Catov JM, Talbott EO, Ritz B (2013). First trimester exposure to ambient air pollution, pregnancy complications and adverse birth outcomes in Allegheny County, PA. Matern Child Health J.

[49] Cai Y, Zhang B, Ke W, Feng B, Lin H, Xiao J (2016). Associations of short-term and long-term exposure to ambient air pollutants with hypertension: a systematic review and meta-analysis. Hypertension.

[50] Vinikoor-Imler LC, Gray SC, Edwards SE, Miranda ML (2012). The effects of exposure to particulate matter and neighbourhood deprivation on gestational hypertension. Paediatr Perinat Epidemiol.

[51] Benjamini Y (2010). Simultaneous and selective inference: current successes and future challenges. Biom J.

[52] Li X, Tan H, Huang X, Zhou S, Hu S, Wang X (2016). Similarities and differences between the risk factors for gestational hypertension and preeclampsia: a population based cohort study in South China. Pregnancy Hypertens.

[53] Di Q, Wang Y, Zanobetti A, Wang Y, Koutrakis P, Choirat C (2017). Air pollution and mortality in the Medicare population. N Engl J Med.

[54] Hoek G, Krishnan RM, Beelen R, Peters A, Ostro B, Brunekreef B (2013). Long-term air pollution exposure and cardio- respiratory mortality: a review. Environ Health.

[55] Kaufman JD, Adar SD, Barr RG, Budoff M, Burke GL, Curl CL (2016). Association between air pollution and coronary artery calcification within six metropolitan areas in the USA (the multi-ethnic study of atherosclerosis and air pollution): a longitudinal cohort study. Lancet.

[56] Szklo M, Nieto J (2012). Epidemiology: beyond the basics 3rd edition Burlington, MA: Jones & Bartlett Learning.

[57] Ananth CV, Keyes KM, Wapner RJ (2013). Pre-eclampsia rates in the United States, 1980-2010: age-period-cohort analysis. BMJ.

[58] Lamminpaa R, Vehvilainen-Julkunen K, Gissler M, Heinonen S (2012). Preeclampsia complicated by advanced maternal age: a registry-based study on primiparous women in Finland 1997-2008. BMC Pregnancy Childbirth.

[59] Kim J-A, Yoon S, Kim L-Y, Kim D-S (2017). Towards actualizing the value potential of Korea health insurance review and assessment (HIRA) data as a resource for Health Research: strengths, limitations, applications, and strategies for optimal use of HIRA data. J Korean Med Sci.

[60] Ghosh G, Grewal J, Männistö T, Mendola P, Chen Z, Xie Y (2014). Racial/ethnic differences in pregnancy-related hypertensive disease in nulliparous women. Ethn Dis.

[61] Xiao J, Shen F, Xue Q, Chen G, Zeng K, Stone P (2014). Is ethnicity a risk factor for developing preeclampsia? An analysis of the prevalence of preeclampsia in China. J Hum Hypertens.

[62] Basso O, Wilcox AJ, Weinberg CR, Baird DD, Olsen J (2004). Height and risk of severe pre-eclampsia. A study within the Danish National Birth Cohort. Int J Epidemiol.

[63] Waterstone M, Bewley S, Wolfe C (2001). Incidence and predictors of severe obstetric morbidity: case-control study. BMJ.

[64] Kang YW, Ko YS, Kim KY, Sung C, Lee DH, Jeong E (2015). Trends in health-related behaviors of Korean adults: study based on data from the 2008 - 2014 community health surveys. Epidemiol Health.

[65] Hong E, Ahn BC (2011). Income-related health inequalities across regions in Korea. Int J Equity Health.

[66] Ye C, Ruan Y, Zou L, Li G, Li C, Chen Y (2014). The 2011 survey on hypertensive disorders of pregnancy (HDP) in China: prevalence, risk factors, complications, pregnancy and perinatal outcomes. PLoS One.

[67] Savitz DA, Danilack VA, Engel SM, Elston B, Lipkind HS (2014). Descriptive epidemiology of chronic hypertension, gestational hypertension, and preeclampsia in New York state, 1995-2004. Matern Child Health J.

[68] Muti M, Tshimanga M, Notion GT, Bangure D, Chonzi P (2015). Prevalence of pregnancy induced hypertension and pregnancy outcomes among women seeking maternity services in Harare, Zimbabwe. BMC Cardiovasc Disord.

[69] Ebeigbe PN, Igberase GO, Aziken ME (2007). Hypertensive disorders in pregnancy: experience with 442 recent consecutive cases in Benin city, Nigeria. Niger Med J.

[70] Jhun HJ, Seo HG, Lee DH, Sung MW, Kang YD, Syn HC (2010). Self-reported smoking and urinary cotinine levels among pregnant women in Korea and factors associated with smoking during pregnancy. J Korean Med Sci.

[71] U.S. Environmental Protection Agency: Integrated science assessment for particulate matter (Report No. EPA/600/R-08/139F). In.: U.S. Environmental Protection Agency, Office of Research and Development, Research Triangle Park, NC.; 2009.

