# Supplementary material for: Exposure to air pollution during preconceptional and prenatal periods and risk of hypertensive disorders of pregnancy: a retrospective cohort study in Seoul, Korea
Source: BMC Pregnancy Childbirth. 2018 Aug 22;18:340. doi: 10.1186/s12884-018-1982-z (PMC6106837; doi:10.1186/s12884-018-1982-z)
Supplement: Supplementary file 1 — Figure S1. Odds ratios and 95% confidence intervals of three types of hypertensive disorders of pregnancy for interquartile range increases in five air pollutant concentrations for 12 months and 1 month before birth by four birth seasons in 18,835 pregnant women residing in Seoul from the Korean National Health Insurance Service–National Sample Cohort for 2002–2013. No particular season showed consistently large risk estimates compared to the other seasons. PM10, particulate matter; NO2, nitrogen dioxide; CO, carbon monoxide, SO2, sulfur dioxide; O3, ozone; GHTN, Gestational hypertension; PE, preeclampsia; Mg-PE, preeclampsia requiring magnesium sulfate. First hollow squares indicate risk estimates not adjusted for birth season. From the first to fourth solid squares in the same color represent spring, summer, fall and winter in order. (DOCX 142 kb) [file 12884_2018_1982_MOESM1_ESM.docx]

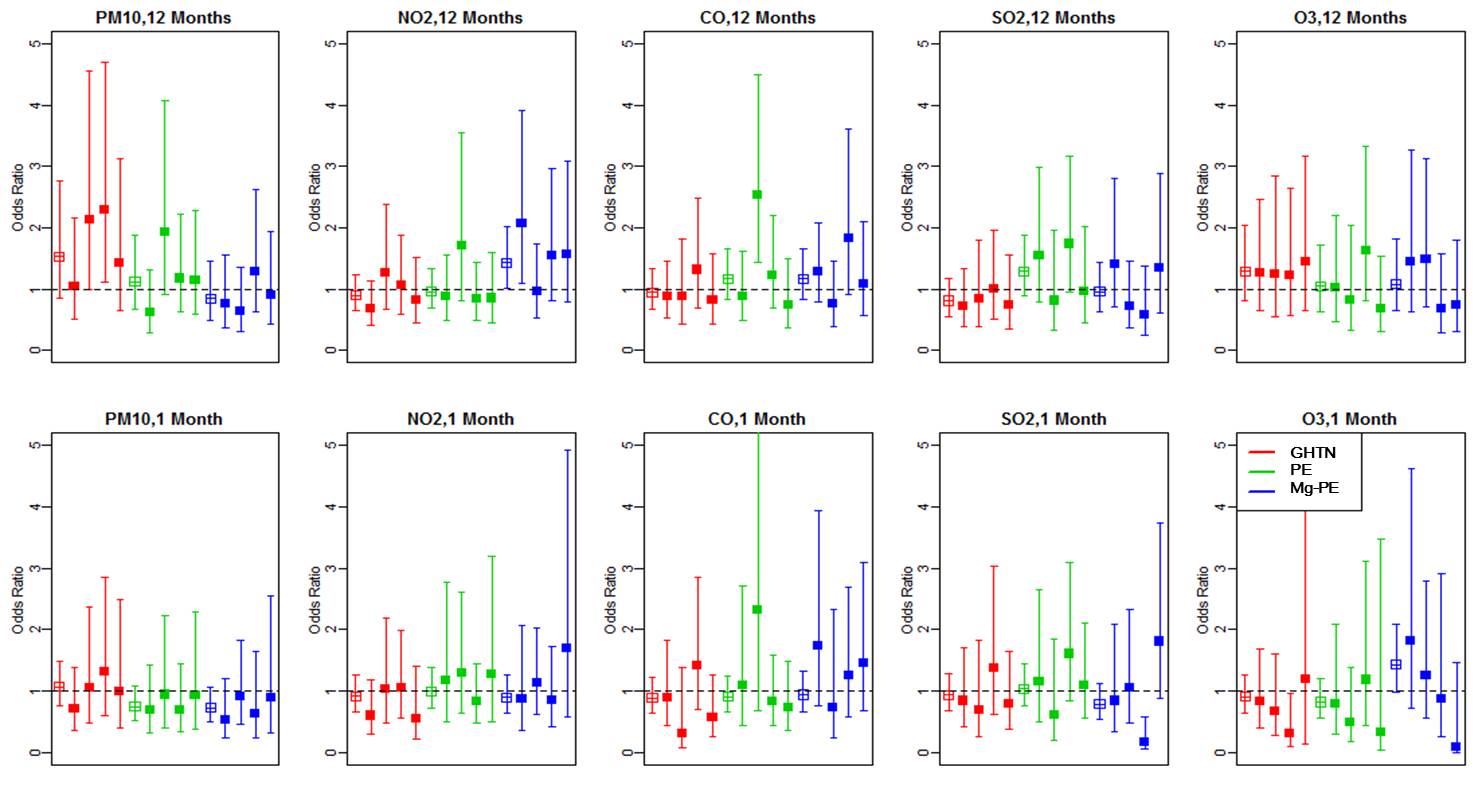


Supplemental figure 1. Sensitivity analysis with stratification of birth season for risk of each hypertensive disorder of pregnancy

PM_10,_ particulate matter; NO_2,_ nitrogen dioxide; CO, carbon monoxide, SO_2,_ sulfur dioxide; O_3,_ ozone; GHTN, Gestational hypertension; PE, preeclampsia; Mg-PE, preeclampsia requiring magnesium sulfate. First hollow squares indicate risk estimates not adjusted for birth season. From the first to fourth solid square in the same color represent spring, summer, fall and winter in order.
