# Supplementary material for: Exposure to air pollution during preconceptional and prenatal periods and risk of hypertensive disorders of pregnancy: a retrospective cohort study in Seoul, Korea
Source: BMC Pregnancy Childbirth. 2018 Aug 22;18:340. doi: 10.1186/s12884-018-1982-z (PMC6106837; doi:10.1186/s12884-018-1982-z)
Supplement: Supplementary file 2 — Figure S2. Odds ratios and 95% confidence intervals of three types of hypertensive disorders of pregnancy for interquartile range increases in five air pollutant concentrations for 12 months and 1 month before birth using different degrees of freedom in adjustment for a temporal trend. Patterns of risk estimates using different degrees of freedom were generally consistent with those of primary analyses. (DOCX 127 kb) [file 12884_2018_1982_MOESM2_ESM.docx]

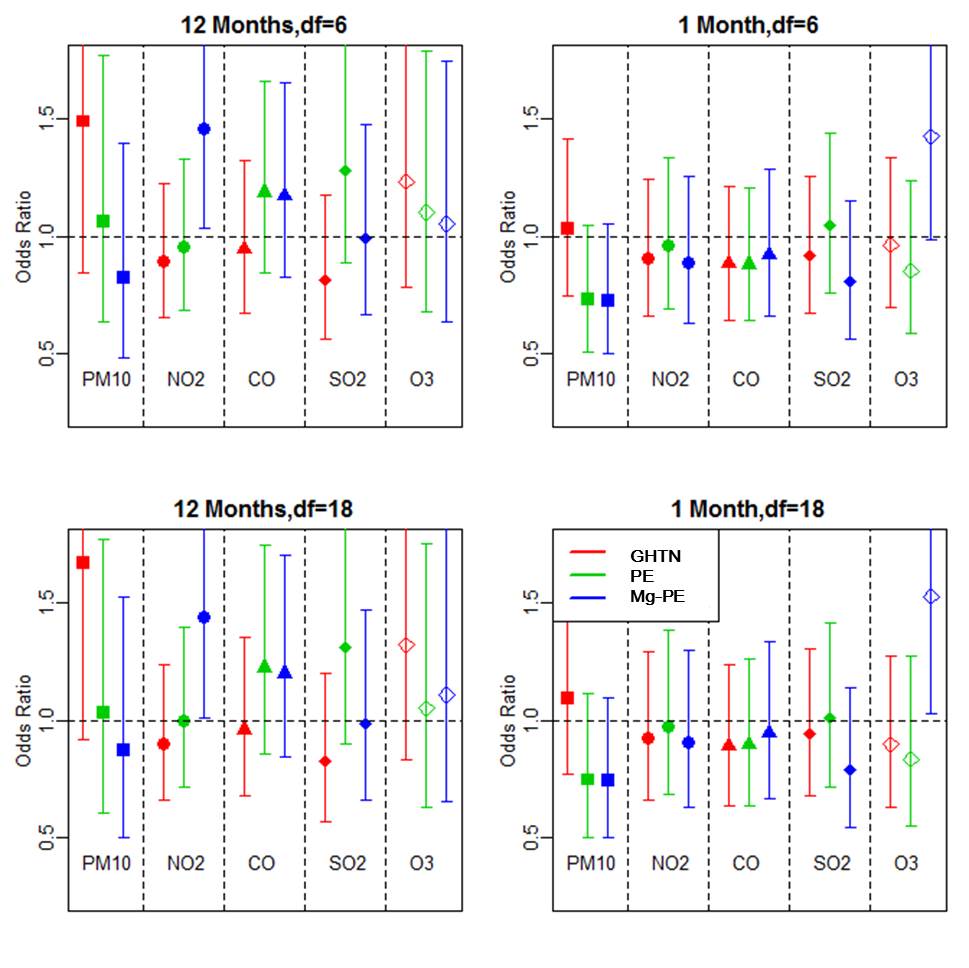


Supplemental figure 2. Additional sensitivity analysis with different degrees of freedom for risk of each hypertensive disorder of pregnancy.

PM_10,_ particulate matter; NO_2,_ nitrogen dioxide; CO, carbon monoxide, SO_2,_ sulfur dioxide; O_3,_ ozone; GHTN, Gestational hypertension; PE, preeclampsia; Mg-PE, preeclampsia requiring magnesium sulfate.
